# Supplementary material for: Harnessing interpretable novel combination of GloVe embedding with deep CNN-BiLSTM neural network for fake news detection
Source: PLoS One. 2025 Sep 22;20(9):e0330154. doi: 10.1371/journal.pone.0330154 (PMC12453182; doi:10.1371/journal.pone.0330154)
Supplement: Attachment — Submitted filename: Authors_Response_on_Reviewers_Comments.pdf [file pone.0330154.s002.pdf]

Response to Reviewers Comments

Manuscript Number: **PONE-D-25-02599**

Journal: PLOS ONE

Title: Harnessing Interpretable Novel Combination of GloVe Embedding With Deep CNN-BiLSTM Neural Network for Fake News Detection

Dear Editor,

Thank you very much for allowing a resubmission of our manuscript, with an opportunity to address the reviewers' comments.

We would like to thank the editor and all the worthy reviewers for their efforts, valuable comments and suggestions. The comments given improved the quality of our paper. Based on the feedback, we have extensively revised our manuscript. The updated manuscript with highlighting indicates changes. The detailed modifications to address reviewers' comments are provided point-by-point in the following.

Sidra khalid et al.

Reviewer Comments to Author

Reviewer: 1

Thank you for inviting me to review this manuscript. This topic is highly relevant in today's age, where the spread of misinformation poses significant challenges to society. Overall, the research appears to be well conducted and provides valuable contributions into improving the accuracy of fake news detection.

However, I have several suggestions that I believe could further strengthen the manuscript and enhance its clarity, comprehensiveness. These suggestions are detailed below.

Author response:

The authors are highly grateful for your efforts and insightful comments. We apologize f or the inconvenience and inappropriate language that raised ambiguity. The paper is exte nsively revised to remove ambiguity.

Point 1: With regard to Section 1, I thought it would be useful to review existing fake news detection techniques in more detail. I feel that a brief summary of existing technologies, including their advantages and disadvantages, would better highlight the need for this study. In addition, although the issue of fake news in the 2016 US election was mentioned, I think it can be added to some new trends in the spread of fake news in recent years, such as the emergence of multimodal fake news, so that readers can better understand the current challenges. In addition, in the contribution summary, I think the contributions and innovations of this study and other similar studies can be compared in more detail. For example, the uniqueness of this study in terms of model performance and interpretability can be highlighted. In addition, although the use of XAI technology to improve the interpretability of the model is mentioned, I think you can explain the argument further.

Author response: As per your valuable suggestion, we have added a brief summary of existing technologies, including their advantages and disadvantages, would better highligh t the need for this study the introduction section in the updated version of the manuscrip t as:

“Researchers have explored many strategies [9] in recent times for the detection of fake news among traditional ML models. ML practitioners use conventional algorithms Suppo rt Vector Machines (SVM), with the help of feature extraction tools, TFIDF, and Bag-of -Words. Such detection methods are easily implementable but provide limited engineerin g features and lack the necessary capacity to understand contextual and semantic linguist ic meaningfulness. Contextual modeling happens automatically in deep learning models, which include Convolutional Neural Network(CNN) and Recurrent Neural Networks(RN N), together with transformer models BERT and RoBERTa that achieve superior perform ance outcomes. Recent studies [10] have investigated combination methods between textu al content and visual components and contextual factors because these approaches show an improved ability to discover fake news on multiple platforms. GloVe embeddings pro

vide a strong framework by partnering with deep CNN-BiLSTM networks to obtain the best attributes from the word context sense, together with deep neural network capability. The deployment of these methods produces successful extraction of syntactic and semant ic elements while providing an interpretable model that addresses current difficulties with most DL systems. The existing detection methods face restrictions that prevent them fro m supporting generalization in different scenarios, handling multilingual content and scar ce resources, and identifying weak or altered material."

As per your valuable suggestion, we have added to some new trends in the spread of fa ke news in recent years the introduction section in the updated version of the manuscript as:

"Fake news proliferation through the internet has reached a critical status because it was exposed during the 2016 U.S. presidential election when misinformation demonstrated its capability to sway massive opinion shifts [2]. After the 2016 U.S. presidential election, f ake information transmission kept maturing through COVID-19 vaccine conspiracies duri ng the Russia-Ukraine conflict, and deceptive disaster accounts about natural disasters. M ultimodal fake news that combines text with images and audio and video elements has b ecome a widespread form of deceptive information since its emergence in recent years. This new version of fake news presents a more compelling, deceptive framework while s imultaneously becoming harder for audiences to detect. The complex environment of fak e news detection has become even more difficult to manage because of deepfakes alongs ide other AI-generated media, which makes existing detection systems less effective."

As per your valuable suggestion, we have enhanced the contribution summary in the intr oduction section in the updated version of the manuscript as:

"Research contributions

The key contributions of this research are:

- The research explores many advanced fake news detection models through FastTe xt-BiLSTM and GloVe with CNN-BiLSTM, LSTM without FastText, and Logisti c Regression with TF-IDF, which are benchmarked using various datasets for ide ntifying their best and worst attributes.
- The GloVe with CNN-BiLSTM model exhibits excellent state-of-the-art capability that surpasses current standards for fake news detection by achieving high precis ion with robust performance.
- The integration of XAI methodology incorporates LIME (Local Interpretable Mod el-agnostic Explanations) technology to make CNN-BiLSTM decision processes m ore understandable, which increases trust in prediction outcomes.
- The main innovative aspect of this research develops a framework that maintains excellent detection accuracy and enhances interpretable models for addressing dua l performance and interpretability requirements in fake news detection systems.
- The approach in this research combines the best features of accuracy and interpre tability, while other studies have usually focused on one aspect. The existing dee p learning approaches for fake news detection achieve high accuracy metrics at th e cost of interpretability. This research distinguishes itself by combining LIME wi

th GloVe and CNN-BiLSTM to deliver high performance with interpretability in fake news detection systems, which aims to establish both effective and trustworthy systems.

- LIME enhances the transparency of CNN-BiLSTM by showing the predictive features while performing its operations within the model. The detection system demands transparency to create trust when applied in genuine fake news scenarios."

As per your valuable suggestion, we have enhanced the XAI technology summary in the introduction section in the updated version of the manuscript as:

"Despite significant advancements in fake news detection using deep learning and NLP, current systems often prioritize accuracy at the expense of interpretability. Moreover, many existing approaches lack the ability to generalize across different languages and platforms. The growing complexity of fake news, particularly through multimodal and AI-generated content such as deepfakes, has further challenged the effectiveness of traditional models. These models typically fail to offer transparency, which is crucial for building user trust in real-world applications."

Point 2. In the introduction of relevant work, that is, Section 2, I think a more systematic classification and summary of the references can be made. For example, work can be divided into several categories, such as machine learning-based methods, deep learning-based methods, and interpretable AI-based methods, and then a brief summary of each category. Also, if you think it's reasonable, I recommend quoting the following jobs that do fake news in the Related Work section: 1. A prompting multi-task learning-based veracity dissemination consistency reasoning augmentation for few-shot fake news detection 2. Fake News Detection on social media: A Data Mining Perspective 3. Veracity-Oriented Context-Aware Large Language Models–Based Prompting Optimization for Fake News Detection

Author response:

As per your valuable suggestion, we have divided the work into several categories in the literature section in the updated version of the manuscript.

In addition, we have discussed your provided related work in in the updated version of the manuscript as:

31. W. Jin, N. Wang, T. Tao, M. Jiang, Y. Xing, B. Zhao, H. Wu, H. Duan, and G. Yang. A prompting multi-task learning-based veracity dissemination consistency reasoning augmentation for few-shot fake news detection. *Engineering Applications of Artificial Intelligence*, vol. 144, p. 110122, 2025.

32. K. Shu, A. Sliva, S. Wang, J. Tang, and H. Liu. Fake news detection on social media: A data mining perspective. *ACM SIGKDD Explorations Newsletter*, vol. 19, no. 1, pp. 22–36, 2017.

Point 3. In the data preprocessing Section in Section 3, I noticed that the author used SMOTE to solve the data imbalance problem. I would like to ask why SMOTE was chosen over other methods, such as undersampling? I think it would be more convincing if I explained the reasons for my choice. Also, in the feature embeddings section, although FastText and GloVe embeddings are mentioned, I thought it would be easier for the reader to add a flow chart

showing how the text is converted into embedded vectors.

Author response:

As per your valuable suggestion, we have added the reasons for using SMOTE in the D ata Balancing section in the updated version of the manuscript as:

"In Contrast with under sampling involves random minority sample removal that could waste important data points, while SMOTE enhances class balance by conserving origina l samples. Through this technique, the model can acquire equivalent information from th e classes without losing important samples. We chose SMOTE over other oversampling methods due to the moderate imbalance in our dataset, as it allowed us to preserve the d iversity of training samples."

In addition, we have added a flowchart in the Feature Embedding Representations sectio n in the updated version of the manuscript as:

"The embedding representation process is illustrated in full detail through Figure 3. This figure demonstrates the tokenization steps that generate numerical input through the pre-t rained word embeddings of FastText and GloVe. The diagram demonstrates a method to handle out-of-vocabulary words through zero vector assignment, which provides consiste nt coverage for every token."

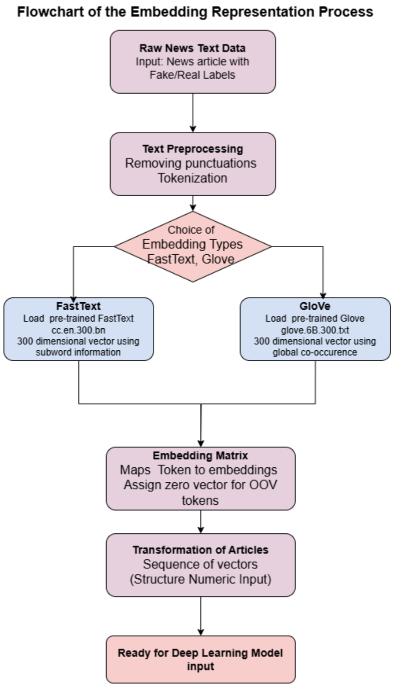

Fig 3. Flowchart of the embedding process converting news text into vectorized input us ing FastText or GloVe for deep learning models.

Point 4. In addition, I think the data set description in Chapter 3 can be supplemented with the sources and collection methods of data sets. This will make the reader more aware of the quality and representativeness of the data. As for the data preprocessing part, you can also discuss the specific preprocessing steps in depth.

Author response:

As per your valuable suggestion, we have revised the dataset description in the updated version of the manuscript as:

"The real news articles were gathered from the Reuters news agency, a globally recogniz ed and credible source for authentic journalism. The information collection process comb ined automated scraping techniques for sorting articles from these sources with manual v erification operations to validate record authenticity. These scholarly articles were publish ed across the years from 2016 to 2017 during which fake news distribution reached its p eak especially in political environments."

Furthermore, we have expanded the data preprocessing section to discuss the specific pre processing steps in greater depth in the updated version of the manuscript as:

"Data Preprocessing

Data preprocessing is crucial in order to prepare the raw text data for embedding generat ion and input to machine learning models. The following detailed steps outline the prepr ocessing methods applied to the dataset:

- Tokenization is done manually, without help from external libraries. It included b reaking a text column down to single words (tokens) by whitespace and punctuati on. Tokenization makes it a structured textual representation of raw text broken d own into smaller analyzable units.
- After tokenization, the sequences of tokens had different lengths. To maintain uni formity and help gain compatibility with deep learning models, all sequences wer e padded to a fixed length of 500 tokens. This was done with the pad sequences method, which adds zeroes in front of shorter sequences. The maximum sequence length (500 tokens) was chosen based on the longest article in the dataset to ens ure that not a single bite of meaningful information was lost up to truncation."

Point 5. In Section 4, in the experimental design section, you chose an 80%-20% data segmentation ratio. I would like to know why this ratio is chosen instead of other ratios, such as 70%-30%? I think it would be better to add the reasons for choosing this ratio. In addition, I suggest that in the model evaluation section, I think it is possible to add an explanation of the meaning and importance of indicators such as accuracy, accuracy, recall and F1 scores.

Author response:

As per your valuable suggestion, we have added the reasons 80%-20% in the experiment al design section in the updated version of the manuscript as:

"The dataset followed stratified sampling to divide itself into (80\%) training data and (2 0\%) testing data. These particular ratios were selected to achieve adequate training data quantity along with a reliable, unbiased evaluation subset. Using a split of (80\%)-(20\%) in machine learning experiments is standard practice when working with datasets contain ing 44,000 records and above, since it provides optimal training and testing performance. A (20\%) test set was utilized throughout the experiment, running on Google Colab, bec ause it decreased resource usage and computation time, yet upheld evaluation standards."

In addition, we have added an explanation of accuracy, recall and F1 scores metrics in the updated version of the manuscript as:

"

Accuracy

The overall performance of the applied model is evaluated by the accuracy metric, which is the ratio of the number of correct predictions to the number of total predictions.

$$\text{Accuracy} = \frac{\text{Number of Correct Predictions}}{\text{Total Number of Predictions}} \tag{1}$$

Precision

The measurement of the accuracy of positive prediction is called precision, also known as positive predictive value. It is the ratio of true positive predictions to the number of positive predictions.

$$\text{Precision} = \frac{\text{Number of Positive Predictions}}{\text{Total Positive Predictions}} \tag{2}$$

Recall

Recall (Sensitivity) measures the ability of the applied model to correctly identify all positive instances. It is the ratio of true positive predictions to the total number of actual positive instances.

$$\text{Recall} = \frac{TP}{TP + FN} \tag{3}$$

F1 Score

The F1 Score is the harmonic mean of Recall and Precision scores, which provides a single metric that balances both the precision and recall of the model, especially useful when the class distribution is imbalanced. This metric usually tells us how precise and robust our applied model is.

$$\text{F1 Score} = \frac{2 \times (\text{Precision} \times \text{Recall})}{\text{Precision} + \text{Recall}} \tag{4}$$

The metrics above were computed from the test data intended to measure how well each of the models has identified fake news.

"

Point 6. In Section 5, the Future Work section, I feel I can describe in more detail the specific application of the advanced models and techniques mentioned. For example, discuss how these models and techniques can be applied to fake news detection and their potential impact.

Author response:

As per your valuable suggestion, we have revised the Future Work in the updated version of the manuscript as:

"Future Work

In the future, fake news detection applications will prove valuable when more efficient models use advanced techniques, such as attention mechanisms, or transformer-based mo dels like BERT or RoBERTa, to understand language with very sophisticated nuances. T he hybrid models developed in this work can be taken towards further perfection by hyp erparameter fine-tuning and including multi-modal data, such as images or videos, to im prove robustness. Increasing the dataset will also extend definition coverage on topics an d timeframes, vastly improving generalizability with models across different domains. Re al-time fake news detection systems need to be deployed through the use of lightweight or compressed deep learning models as a valuable direction for implementing the detecti on systems. A range of tools, including browser extensions, APIs, and integration within social media platforms, could serve as deployments to stop the spread of misinformation on a large scale."

Point 7. Overall, the manuscript presents a thorough investigation into leveraging advanced deep learning models and explainable AI techniques for FND area. Given the importance of the topic and the potential impact of the research, I recommend a Major Revision to address the detailed suggestions provided in this review.

Author response:

We again apologize for the inconvenience and inappropriate language that raised ambigui ty. The updated paper is extensively revised to remove ambiguity.

Reviewer: 2

I congratulate the authors for their contribution and hard work. Before going to publish the manuscript, a few perhaps would get clarified from my end.

Point 1. In this work, the authors proposed comparative analysis of innovative hybrid deep learning models and embedding techniques-focusing interpretability using Xplainable Artificial Intelligence (XAI) for fake news detection. Elaborate, how it is better compared to the existing methods. Provide more clarifications.

Author response:

The authors are highly grateful for your efforts and insightful comments. We apologize f or the inconvenience and inappropriate language that raised ambiguity. The paper is exte nsively revised to remove ambiguity.

Elaboration:

Our proposed GloVe-Based CNN-BiLSTM model achieves an accuracy of 99.63%, outpe rforming existing methods such as FakeBERT (98.90%), BiLSTM with self-attention (98. 65%), and Multitask learning with CapsNet and BiLSTM (97.96%). The combination of GloVe embeddings, CNN, and BiLSTM enables the model to effectively capture both se

mantic and contextual dependencies, significantly enhancing its performance.

In addition to superior accuracy, our model integrates Explainable Artificial Intelligence (XAI) techniques, including attention visualization and relevance propagation, which prov ide crucial interpretability. This transparency allows users to understand the model's deci sion-making process, addressing the limitations of traditional "black-box" models and inc reasing trust in real-world applications such as fake news detection.

In summary, our model not only delivers improved accuracy but also offers interpretabili ty, making it a notable advancement over existing methods in fake news detection.

Point 2. Add more recent references and compare proposed method with Existing works.

Author response:

As per your valuable suggestion, we have revised the Existing Work by Add more recen t references and comparison in the updated version of the manuscript as:

"State of the art Comparison

The GloVe-Based CNN-Bidirectional LSTM Model has achieved the highest accuracy of 99.63\% in fake news detection, which surpasses all current state-of-the-art methods. Wh en compared to FakeBERT (98.90\%), BiLSTM with self-attention layers (98.65\%), and Multitask learning with CapsNet and BiLSTM (97.96\%), it is quite evident that this mo del outperforms its predecessors in detecting fake news. Another recent model, AugFake-BERT, achieved 92.45\% accuracy for fake news classification. Table \ref{tabsota} show s that the success of this model is due to the impactful feature representation by the po werful GloVe embedding along with the robust CNN and BiLSTM architectures, which can pick up both the semantic and contextual dependencies present in the text. The resul ts confirmed the power of the present model in bringing forth realistic accuracy and relia bility in fake news detection, and thus, it adds significantly to this field."

Table 8. State of the art comparison.

Ref	Proposed Technique	Accuracy
[16]	BERT + CNN for fake news detection	98.90%
[17]	BiLSTM with self-attention layers	98.65%
[18]	Multitask learning with CapsNet and BiLSTM	97.96%
[22]	AugFake-BERT	92.45%
**Proposed**	**Glove Based CNN-BILSTM Model**	**99.63%**

Point 3. The paper is technically sound but need to improve the grammar.

Author response:

We again apologize for the inconvenience and inappropriate language that raised ambigui ty. The paper is extensively revised to remove ambiguity and to improve the grammar.

Point 4. Provide more relevant information regarding the figures.

Author response:

As per your valuable suggestion, we have revised the details regarding figures in the up

dated version of the manuscript. Each figure now includes a clearer description of its significance, the data it represents, and how it supports the findings of the study. We believe these additions will enhance the clarity and context of the figures, making them more informative for readers.

Point 5. The alignment of data available in figures 7 and 8 are not visible to identify the fake news.

Author response:

We have corrected the alignment and improved the clarity of these figures to ensure that the data is clearly visible and properly aligned for identifying fake news. The revised figures now provide a more accurate representation of the data, making it easier for readers to interpret and understand the results as:

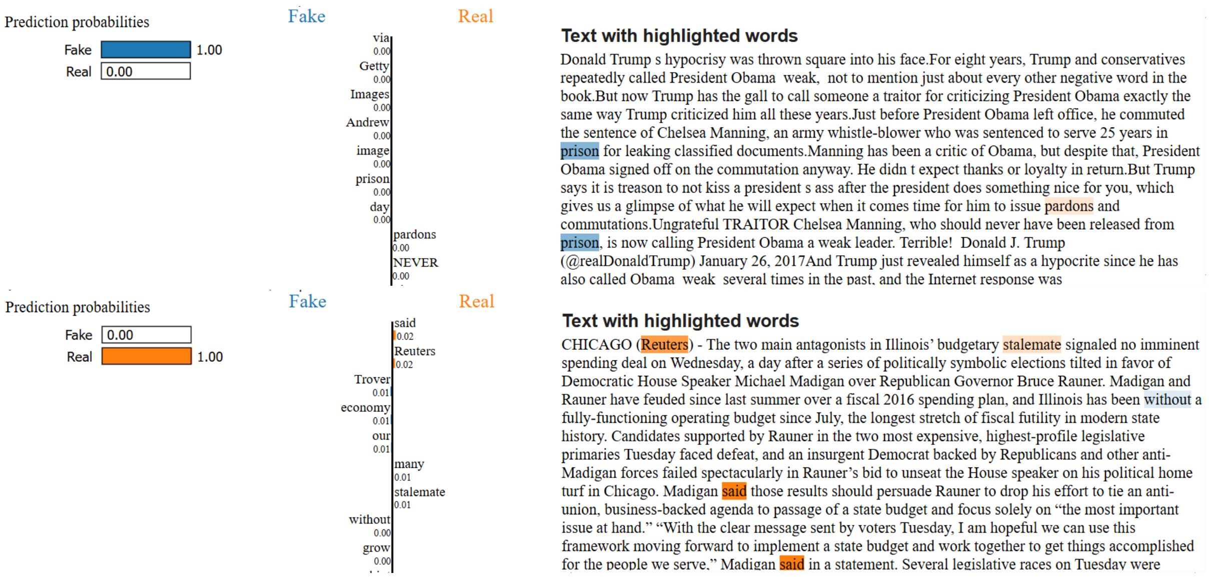

Point 6. Polish the Language.

Author response:

We again apologize for the inconvenience and inappropriate language that raised ambiguity. The paper is extensively revised to remove ambiguity and to improve the grammar.

Point 7. Instead of Tables, Compare the Existing and proposed methods using Bar Graphs to prove proposed method is more Superior.

Author response:

As per your valuable suggestion, we have added a bar graphs to visually compare the performance of existing and proposed methods in the updated version of the manuscript as:

"An illustration, Figure 10, through a bar chart, reveals the comparison between existing and proposed modern fake news detection methodologies. The proposed Glove-based CNN-Bilstm model achieves the highest accuracy of 99.63\% as it demonstrates superiority over existing methods."

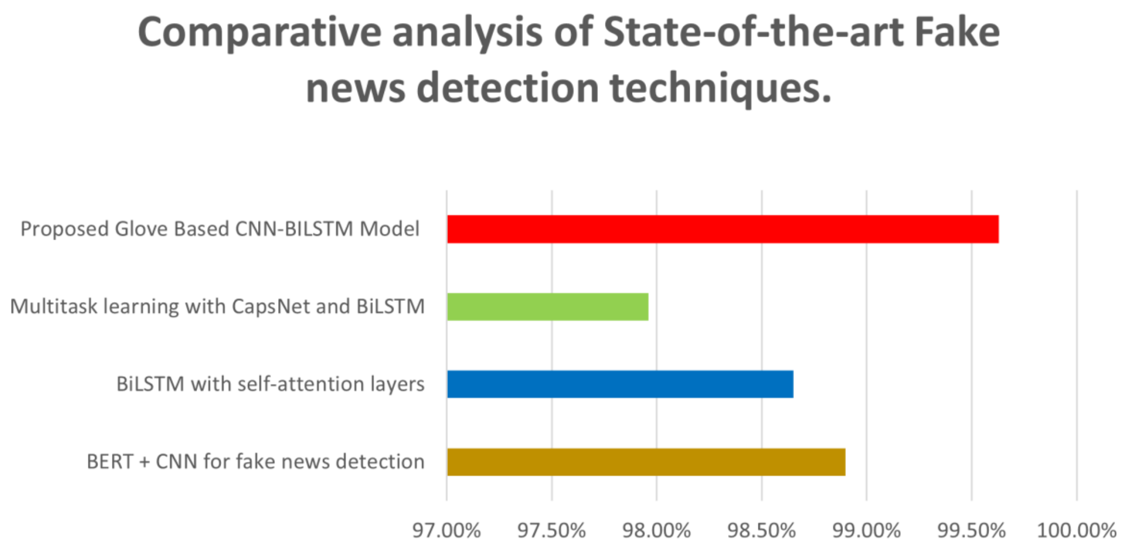

Point 8. Problem statement is not clearly mentioned.

Author response:

As per your valuable suggestion, we have clearly the problem statement in the introduction section in the updated version of the manuscript as:

"Problem Statement

Despite significant advancements in fake news detection using deep learning and NLP, current systems often prioritize accuracy at the expense of interpretability. Moreover, many existing approaches lack the ability to generalize across different languages and platforms. The growing complexity of fake news, particularly through multimodal and AI-generated content such as deepfakes, has further challenged the effectiveness of traditional models. These models typically fail to offer transparency, which is crucial for building user trust in real-world applications."

The updated version now explicitly defines the challenges in fake news detection, the limitations of existing methods, and the motivation for our proposed approach. This revision aims to provide a clearer understanding of the research problem and the significance of our work.

Point 9. Related work needs to be improved.

Author response:

As per your valuable suggestion, we have completely revised the Related Work section to provide a more comprehensive overview of the current state of research in fake news detection. We have included additional relevant studies, highlighted key advancements, and clearly positioned our proposed method in relation to existing approaches. This improvement aims to offer a more thorough context for our work, showcasing its novelty and contributions to the field.

Point 10. The Sections are not aligned properly.

Author response:

As per your valuable suggestion, we have carefully reviewed the entire manuscript and r ealigned all sections to maintain a consistent and logical structure in the updated version of the manuscript. This revision enhances the overall presentation and ensures a clearer a nd more organized flow of content throughout the paper.
