# Supplementary material for: Harnessing interpretable novel combination of GloVe embedding with deep CNN-BiLSTM neural network for fake news detection
Source: PLoS One. 2025 Sep 22;20(9):e0330154. doi: 10.1371/journal.pone.0330154 (PMC12453182; doi:10.1371/journal.pone.0330154)
Supplement: Attachment — Submitted filename: Authors_Response_on_Reviewers_Comments_auresp_2.pdf [file pone.0330154.s003.pdf]

Response to Reviewers Comments

Journal: PLOS ONE

Title: Harnessing Interpretable Novel Combination of GloVe Embedding With Deep CNN-BiLSTM Neural Network for Fake News Detection

Dear Editor,

Thank you very much for allowing a resubmission of our manuscript, with an opportunity to address the reviewers' comments.

We would like to thank the editor and all the worthy reviewers for their efforts, valuable comments and suggestions. The comments given improved the quality of our paper. Based on the feedback, we have extensively revised our manuscript. The updated manuscript with highlighting indicates changes. The detailed modifications to address reviewers' comments are provided point-by-point in the following.

Khalid et al.

Reviewer Comments to Author

Additional Editor Comments:

The two anonymous reviewers have proposed several suggestions and both of them think you should proof your language skills and your writing to make your paper easy-following. So please complete and solve their concerns in the next two months.

Author response:

The authors are highly grateful for your efforts and insightful comments. We apologize f or the inconvenience and inappropriate language that raised ambiguity. The paper is exte nsively revised to remove ambiguity.

Reviewer: 1

Although this paper has been reviewed by the other reviewer in the last round, but I also found some issues that the author should pay attention to, which are listed as follows:

1. The state-of-the-art comparison (Table 8) needs updating with more recent works (2023-2024) to properly contextualize the claimed novelty. The field evolves rapidly.

Author response:

The authors are highly grateful for your efforts and insightful comments. We apologize f or the inconvenience and inappropriate language that raised ambiguity. The paper is exte nsively revised to remove ambiguity.

As per your valuable suggestions, we have updated Table 9 in the manuscript to include several recent state-of-the-art approaches published in 2023, 2024, 2025 as:

Table 8. State of the art comparison.

Ref	Proposed Technique	Accuracy
[14]	BERT + CNN for fake news detection	98.90%
[16]	BiLSTM with self-attention layers	98.65%
[17]	Multitask learning with CapsNet and BiLSTM	97.96%
[21]	AugFake-BERT	92.45%
[33]	Courtroom-FND	78.60%
[34]	CAPE-FND	81.20%
**Proposed**	**Glove Based CNN-BILSTM Model**	**99.63%**

33. Jin W, Su D, Tao T et al. Courtroom-FND: a multi-role fake news detection method based on argument switching-based courtroom debate. *Journal of King Saud University Computer and Information Sciences* 2025; 37(3): 1–29.

34. Jin W, Gao Y, Tao T et al. Veracity-Oriented Context-Aware Large Language Models–Based Prompting Optimization for Fake News Detection. *International Journal of Intelligent Systems* 2025; 2025(1):5920142.

2. The LIME explanations (Figures 8-9) require much more detailed interpretation. What do the highlighted words indicate about the model's decision process? How representative are these examples?

Author response:

As per your valuable suggestions, we have added more detailed interpretation in the man uscript as:

"The LIME analysis in Figure 8 indicates that the article is correctly labelled as Real, w ith a confidence score of 1.00. The words "Reuters", "Trover", "economy", and "stalemat e" are flagged by LIME as the main factors behind the prediction. They appear in politic al, financial, and official settings, which are key parts of real journalism.

The LIME analysis in Figure 9 shows how the audience understands a fake news article about the previous U.S. President Donald Trump's politically charged remarks. Accordin g to the model, the article is Fake with a probability of 100 percent. LIME brings out th e words "NEVER", "traitor", "prison", and "piece", pointing out that this type of langua ge tends to be loaded with emotion, offers opinions, and uses strong words that are typi cal in misinformation or editorials."

3. The dataset section should address potential source biases (e.g., PolitiFact's political leanings) and how this might affect model generalizability.

Author response:

As per your valuable suggestions, we have updated dataset section in the manuscript as:

"The fake news data were mainly taken from PolitiFact and Wikipedia, and the real new s came from Reuters, a famous traditional news outlet worldwide. PolitiFact, despite bein g trustworthy, may exhibit small biases in its decisions to call out fake content, dependin g on what journalists consider true or false. Similarly, relying solely on Reuters for news may lead the model to perform well in specific styles or situations, but not necessarily i n all cases. We employed preprocessing techniques to guide the model in focusing on la nguage aspects rather than the unique characteristics of each country."

4. The methodology needs clearer justification for architectural choices (e.g., why 128 units in BiLSTM? Why 5 epochs?) and hyperparameter tuning process.

Author response:

As per your valuable suggestions, we have added the justification for architectural choice in the manuscript as:

“Hyperparameters Selection

In research experiments, the selection of hyperparameters for each model is based on pri or studies and iterative testing. For applied deep learning models, FastText-BiLSTM, Glo Ve-CNN-BiLSTM, and LSTM, we conducted manual tuning by observing validation acc uracy and loss. We performed careful manual tuning using validation performance as a g uide. The final selected hyperparameters are summarized in Table 6. The key hyperpara meters are chosen due to the following reasons:

- Number of LSTM/CNN units: 64, 128, and 256 were tested; 128 units yielded th e best performance.
- Embedding dimension: Fixed at 300 using pre-trained GloVe and FastText embed dings.
- Batch size: 32, 64, and 128 were evaluated; 64 led to stable convergence.
- Epochs: Capped at 5 based on early stopping to avoid overfitting.
- Optimizer: Adam optimizer was chosen for its adaptive learning rate and consiste nt results.
- Learning rate: Default setting of 0.001 was retained as further tuning did not yiel d significant improvements.
- Loss function: Binary Crossentropy for all models due to the binary classification task.
- Validation split: 20\% of training data was reserved for validation during model t uning.”

Table 6. Key hyperparameters for deep learning models.

Model	Embedding	Units	Epochs	Batch Size	Optimizer	LR	Val. Split
FastText-BiLSTM	FastText (300d)	128 LSTM	5	64	Adam	0.001	20%
GloVe-CNN-BiLSTM	GloVe (300d)	128 LSTM + 64 CNN	5	64	Adam	0.001	20%
LSTM	GloVe (300d)	128 LSTM	5	64	Adam	0.001	20%

5. Moreover, I suggest the author cite the following literature and work in the Related Work section: 1. Fake news detection: comparative evaluation of BERT-like models and large language models with generative AI-annotated data https://arxiv.org/abs/2412.14276 2. Veracity-Oriented Context-Aware Large Language Models–Based Prompting Optimization for Fake News Detection 3. Courtroom-FND: a multi-role fake news detection method based on argument switching-based courtroom debate 4. A prompting multi-task learning-based veracity dissemination consistency reasoning augmentation for few-shot fake news detection.

Author response:

As per your valuable suggestions, we have update related work with your provided refer ence in the literature in the manuscript as:

33. Jin W, Su D, Tao T et al. Courtroom-FND: a multi-role fake news detection method based on argument switching-based courtroom debate. *Journal of King Saud University Computer and Information Sciences* 2025; 37(3): 1–29.

34. Jin W, Gao Y, Tao T et al. Veracity-Oriented Context-Aware Large Language Models–Based Prompting Optimization for Fake News Detection. *International Journal of Intelligent Systems* 2025; 2025(1):5920142.

35. Raza S, Paulen-Patterson D, Ding C. Fake news detection: comparative evaluation of BERT-like models and large language models with generative AI-annotated data. *Knowledge and Information Systems* 2025; 1–26.

6. An error analysis section should be added - what types of fake news still evade detection? Where do the models consistently fail?

Author response:

As per your valuable suggestions, we have added the error analysis section in the manus cript as:

“Error Analysis

The proposed model demonstrates high performance, achieving an accuracy above 99\%. However, there remains a margin of error of approximately 1\%. A detailed error analysi s reveals that some misclassified instances include deceptive articles that mimic the tonal and linguistic style of legitimate news sources. These articles often use formal language, maintain an objective tone, and include credible-sounding named entities, which can cau se the model to mistakenly classify them as genuine. Such articles may also embed misl eading narratives within seemingly factual claims, which even confuse ensemble classifie rs. Additionally, some honest articles were incorrectly flagged as fake. These were often conspiracy-related or covered controversial topics, frequently featuring sensational or emo tionally charged language, which likely contributed to the misclassification.

Another challenge arises when the model is presented with very short news snippets or h eadlines that lack sufficient context. Due to the limited text, models—especially those rel ying on word embeddings-struggle to extract meaningful semantic features, resulting in c lassification errors.”

7. The model architectures lack sufficient detail about hyperparameter selection and tuning processes. Please introduce the key hyper-parameters in the update experiments section.

Author response:

As per your valuable suggestions, we have added the justification for architectural choice in the manuscript as:

“Hyperparameters Selection

In research experiments, the selection of hyperparameters for each model is based on pri

or studies and iterative testing. For applied deep learning models, FastText-BiLSTM, Glo Ve-CNN-BiLSTM, and LSTM, we conducted manual tuning by observing validation acc uracy and loss. We performed careful manual tuning using validation performance as a g uide. The final selected hyperparameters are summarized in Table 6. The key hyperpara meters are chosen due to the following reasons:

- Number of LSTM/CNN units: 64, 128, and 256 were tested; 128 units yielded th e best performance.
- Embedding dimension: Fixed at 300 using pre-trained GloVe and FastText embed dings.
- Batch size: 32, 64, and 128 were evaluated; 64 led to stable convergence.
- Epochs: Capped at 5 based on early stopping to avoid overfitting.
- Optimizer: Adam optimizer was chosen for its adaptive learning rate and consiste nt results.
- Learning rate: Default setting of 0.001 was retained as further tuning did not yiel d significant improvements.
- Loss function: Binary Crossentropy for all models due to the binary classification task.
- Validation split: 20\% of training data was reserved for validation during model t uning."

Table 6. Key hyperparameters for deep learning models.

Model	Embedding	Units	Epochs	Batch Size	Optimizer	LR	Val. Split
FastText-BiLSTM	FastText (300d)	128 LSTM	5	64	Adam	0.001	20%
GloVe-CNN-BiLSTM	GloVe (300d)	128 LSTM + 64 CNN	5	64	Adam	0.001	20%
LSTM	GloVe (300d)	128 LSTM	5	64	Adam	0.001	20%

8. Moreover, I find that the important metrics like ROC-AUC are missing, which are particularly valuable for evaluating performance on imbalanced data.

Author response:

As per your valuable suggestions, we have included the ROC-AUC score in the manuscr ipt as:

Table 8. The class-wise performance analysis.

Model	Class	Precision	Recall	F1-Score	ROC-AUC
FastText + BiLSTM	Fake News (0)	0.96	0.95	0.96	0.9710
	Real News (1)	0.95	0.96	0.95	
LSTM w/o FastText	Fake News (0)	0.98	0.99	0.98	0.9710
	Real News (1)	0.99	0.98	0.98	
Logistic Regression w TF-IDF	Fake News (0)	0.99	0.99	0.99	0.9989
	Real News (1)	0.99	0.99	0.99	
GloVe + CNN-BiLSTM	Fake News (0)	1.00	0.99	1.00	0.9989
	Real News (1)	0.99	1.00	1.00	

9. The language needs significant polishing throughout - many awkward phrasings and grammatical errors. I suggest the authors should make some language proof serives to make their paper become more easy-understanding.

Author response:

We again apologize for the inconvenience and inappropriate language that raised ambigui ty. The paper is extensively revised to remove ambiguity.

Reviewer: 2

The authors have addressed all the Comments thoroughly except the Language. Try to Polish the Language.

Author response:

The authors are highly grateful for your efforts and insightful comments. We apologize f or the inconvenience and inappropriate language that raised ambiguity. The paper is exte nsively revised to remove ambiguity.
